# Supplementary figures and images for: Synchrotron scanning reveals the palaeoneurology of the head-butting Moschops capensis (Therapsida, Dinocephalia)
Source: PeerJ. 2017 Aug 10;5:e3496. doi: 10.7717/peerj.3496 (PMC5554600; doi:10.7717/peerj.3496)

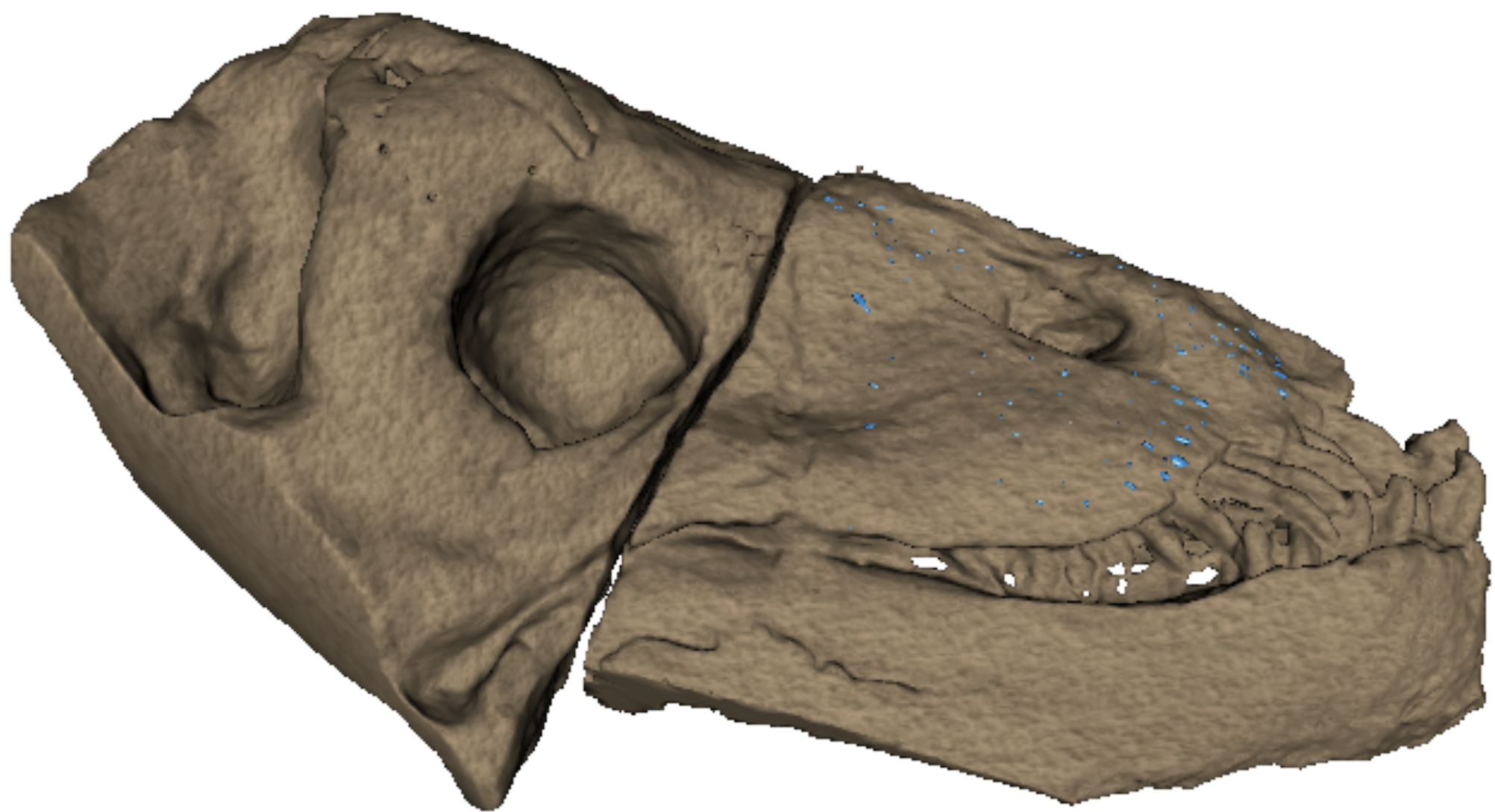

Supplement: Supplemental Information 3 [file peerj-05-3496-s003.pdf]
